# Exploring core concepts and principles underpinning prosthetic and orthotic practice – a qualitative study involving Swedish clinicians

**DOI:** 10.1186/s12909-026-09857-6

**Published:** 2026-07-06

**Authors:** Nerrolyn Ramstrand, Jessica Crafoord, Louise Bæk Larsen, David Rusaw

**Affiliations:** Department of Rehabilitation, School of Health and Welfare, Jönköping University, Box 1026, Jönköping, 551 11 Sweden

**Keywords:** Concept-based education, Assistive technology, Prosthetic limb, Orthotic device, stimulated recall

## Abstract

**Background:**

Concept-based curricula are increasingly being adopted in medical and allied health education, supporting students to organise their knowledge and transfer their learning to unfamiliar situations. The approach is grounded in the professional reasoning and practice underlying specific disciplines and requires detailed knowledge of the core concepts and principles relevant to professional practice. This study focused on the allied health discipline of prosthetics and orthotics and aimed to identify core concepts and principles applied by Swedish-certified prosthetists/orthotists during routine clinical consultations.

**Methods:**

Ten Prosthetists/orthotists with a minimum of 10 years experience were fitted with a chest-mounted video camera which recorded them during routine client consultations. Video recordings were subsequently used in stimulated recall interviews, during which participants watched the footage and were prompted to reflect on, and articulate, thoughts, decision-making processes and feelings experienced at the time of the consultation. Interview transcripts were transcribed verbatim and analysed using inductive thematic analysis to identify core concepts and underpinning principles used by clinicians.

**Results:**

Eleven core concepts were identified and organised under four overarching themes: (1) clinical relationships and systems, (2) clinical evaluation, (3) biomechanics and human movement, and (4) device design, materials, and context. Each core concept was underpinned by between two and eight core principles that explained how clinical reasoning and actions were enacted in practice.

**Conclusions:**

The core concepts and principles identified in this study provide an exploratory practice-informed foundation for the continued development of a comprehensive conceptual framework for the prosthetic and orthotic profession. Findings have the potential to inform concept-based curriculum design, teaching strategies, and assessment approaches, supporting the alignment of educational programs with authentic clinical practice.

**Supplementary Information:**

The online version contains supplementary material available at 10.1186/s12909-026-09857-6.

## Background

Prosthetics and orthotics is an allied health discipline concerned with the assessment, design, and provision of prosthetic limbs and orthotic devices to support mobility, function, and participation. Prosthetic and orthotic curriculum development has traditionally been guided by expert opinion or consensus documents, most commonly articulated through professional profiles [1], core competency descriptions [2–4] and standards documents [4–7]. Although these provide a valuable overview of the factual knowledge and fundamental skills necessary to be an effective clinician, they tend to emphasise observable tasks and roles rather than the underlying structure of disciplinary knowledge. In a shift away from curricula organised around content areas or competencies, toward an emphasis on the “big ideas” that structure and connect professional knowledge, there has been a growing shift among educators toward a core concepts approach to curriculum design, teaching and learning [8–12].

Core concepts have been defined as the broad, enduring ideas that lie at the heart of a discipline. They are typically one- or two-word nouns or short phrases that are “timeless, universal, abstract and exhibit a higher level of abstraction than content topics” [12, 13]. Core concepts are distinct from core competencies, which refer to the fundamental skills necessary to be an effective clinician, in that they describe the underlying constructs that organise and connect knowledge into coherent structures (8, 11). Etukakpan et al. [13] suggest that core concepts serve as cognitive anchors for students, allowing them to integrate new knowledge into their existing mental frameworks. Embedded within and across core concepts are core principles, defined by Chen et al. [11] as smaller foundational ideas that underpin one or more core concepts.

Clear delineation of the terms core principles from core concepts has been complicated by inconsistent terminology in academic and educational literature, with “core concepts” and “core principles” often used variably and, in some cases, synonymously [14, 15]. In the present study, we choose to define core principles as the building blocks of core concepts, encompassing the rules or explanatory mechanisms that describe why phenomena occur and how underlying processes operate in professional practice [11].

A concept-based approach to curriculum design draws on the principle of conceptual learning and reframes educational planning around fundamental, discipline-defining concepts and principles rather than around an extensive list of topics, discrete skills, or task-based competencies [8, 12]. The approach is intended to support learners in identifying relevant knowledge and integrating key ideas so they can apply learning across different situations and contexts [12, 14]. Given that concept-based learning approaches help students to organise relevant knowledge and apply it in unfamiliar situations, it is particularly well-suited to health education, where learners must navigate complex health systems and increasingly difficult patient cases [12].

Discipline-specific core concepts and principles are typically identified through descriptive survey studies and qualitative focus groups with domain educators or experts [11, 13, 16]. Although these methods yield useful and interesting results, both are limited by a reliance on participants’ ability to consciously articulate their thinking, often in artificial or decontextualised settings that remove them from the environment in which the professional activities usually occur. One compelling alternative, previously showing promise in examining the knowledge base of teachers [17, 18], primary care physicians [19] and nursing mentors [20] is stimulated recall. Stimulated recall is a method that was initially inspired by Dewey’s reflecting thinking concept [21], and involves the use of prompts, such as notes or audio and video recordings, to evoke memories of a scenario previously experienced by participants. The data collection process typically begins by capturing stimulus data that exhibits participants’ behaviours or cognitive tasks within the context of interest. This is followed by an individual interview, where the stimulus data is presented back to participants and they are prompted to remember and articulate intrinsic thoughts, mental processes and feelings experienced at the time the stimulus was generated [18]. In an extension of the method, it has been suggested that stimulus data collected from the ‘own point-of-view perspective’ (e.g. body-mounted camera) facilitates a deeper level of recall of cognitive and pre-verbal processes [22].

The aim of this study was to use a qualitative, stimulated recall method to identify the core concepts and principles applied by Swedish-certified prosthetist/orthotists during routine clinical encounters with their clients. By grounding the analysis in clinicians’ real-time practice, it was anticipated that the approach would provide an empirically anchored representation of their professional reasoning, rather than one derived solely from expert opinion or curricular sources. It was intended that the findings would serve as a robust starting point for the continued development of a comprehensive, practice-informed set of core concepts and principles for the prosthetic and orthotic profession.

## Methods

### Research paradigm

This qualitative study was grounded in a constructivist paradigm, which assumes that knowledge and meaning are co-constructed through social interaction and interpretation [23]. The intent of the research was to explore and interpret the core concepts and principles that prosthetic and orthotic clinicians draw upon during clinical consultations. A constructivist approach was considered appropriate as it recognises that application of specific concepts and principles are shaped through clinicians’ experiences, contexts, and interpretation of clinical situations.

A video-assisted stimulated recall method was selected to enable clinicians to reflect on their own client encounters and articulate clinical reasoning that may not be evident in real-time observations [18, 24, 25] or survey questions [26]. Recall sessions were analysed using latent thematic analysis [27]. Through abstraction, core concepts and principles were inferred from clinicians’ descriptions of their clinical reasoning.

### Context and sampling

Data were collected in Swedish prosthetic and orthotic (P&O) facilities, providing a real-world context to explore how core concepts and principles are applied in naturalistic settings. Sweden operates a universal healthcare system, with P&O services funded through regional healthcare budgets [28]. Within this system, P&O facilities function either as public units, typically affiliated with regional hospitals, or as private providers, who compete through a tender system to deliver services under regional healthcare contracts.

Participants were certified prosthetists and orthotists who met the following inclusion criteria: a minimum of 10 years of professional experience, actively involved in client consultations, and current employment in a clinical capacity. These criteria were selected to focus on highly experienced practitioners who were assumed to represent the skills and approaches that educational programs aim to develop in students. Potential participants were excluded if they held a leadership or administrative position and did not have their own patient load.

Clinicians were purposefully sampled to capture variation across clinical specialisations, geographical regions, and facility types (public and private). This ensured a rich and diverse dataset reflective of the range of P&O practices in Sweden. Potential participants were invited via email from the research team. All participants were graduates of the only P&O educational program in Sweden, although they had completed different iterations of the curriculum over time. Participating clinicians were requested to provide informed written consent prior to the study. Patients and staff in the clinic who were captured on footage collected by the GoPro camera were also informed about the study and requested to provide written informed consent. Ethical approval for the study was obtained from the Swedish National Ethics Committee (Dnr 2024-01156-01) and include approval to record GoPro footage and conduct recall sessions.

### Author characteristics and reflexivity

The researchers involved in this study are certified prosthetists and orthotists employed at a Swedish university. Two of the researchers were educated at this same university (JC & LL), while the remaining two completed their professional training in Australia (NR) and Canada (DR), respectively. Two members of the research team conducted the data collection with participants (JC & NR). One of these researchers has been employed at the university for over 20 years and had previously taught several of the participants. The second researcher had been a classmate of one participant. These prior relationships were acknowledged as a potential source of bias, as participants may have modified their responses based on perceived expectations.

All four researchers were involved in data analysis. As each work within the context of P&O education, there was potential for their own expectations of core concepts and principles to influence data interpretation. To mitigate this, reflexivity was maintained through regular data analysis meetings in which the team critically reflected over their assumptions, potential biases, and how these might shape coding and theme development.

### Data collection methods

Participating clinicians were fitted with a chest-mounted GoPro HERO 11 camera (GoPro, Inc, San Mateo, CA, USA), which recorded video and audio during a clinical consultation with a client. To capture a range of prosthetic and orthotic services, researchers purposively selected client experiences to be recorded. This ensured that the selection included consultations with new clients who were meeting the clinician for the first time as well returning clients who were being fit with new devices or were experiencing issues with their current device. The researchers were not present while the clinicians were consulting their client.

Immediately after the client consultation, clinicians met with the researchers for a recall session. During this session clinicians watched the film that had just been captured and were requested to think aloud’, expressing thoughts and feelings that they recalled having at the time of the consultation. Throughout the recall session the researchers would pause the film and ask clinicians to elaborate on their thoughts by using prompts, ”what were you thinking here”, ”can you remember why you decided to do X?”, How did you feel when the client said Y?” Recall sessions were recorded and transcribed verbatim for analysis. All sessions were conducted in Swedish.

### Data analysis

Recall transcripts were analysed using the six-step approach recommended by Braun and Clarke [27]. Coding was conducted in Swedish and facilitated by using NVivo 14 software (QSR Int, Melbourne, Australia). An inductive approach was adopted, beginning with repeated readings of several transcripts to develop a deep understanding of the content.

To support analytic consistency and transparency during abstraction, the research team first developed a shared working definitions of *core concepts* and *core principles*. These definitions were informed by those documented by McFarland and Michaelsen [29] and Schaefer and Michael [8] and were agreed upon by the author group. The resulting definitions with explanatory criteria, are presented in Table 1 and were used to guide decisions about the level of abstraction throughout the analysis. Core concepts were defined as broad, unifying constructs that integrate multiple principles and guide reasoning or action across contexts, while core principles were defined as foundational explanatory ideas underpinning one or more core concepts.

**Table 1:** Definitions of core concepts and principles operationalised by the research team

Feature / Criteria	Core Concept	Core Principle
Purpose	Central to understanding phenomena within clinical and technical P&O practices; integrates multiple principles and guides reasoning or actions to be taken.	Foundational idea that explains *why* phenomena occur; underpins one *or more* core concepts.
Scope	Broad and unifying; applies across a variety of clinical areas, populations, and device types.	Narrower but generalisable; applies in multiple contexts where the principle is relevant.
Endurance	Remains relevant over time because it organizes enduring principles into a framework for reasoning and decision-making.	Remains relevant over time because it describes fundamental, mechanistic truths about how systems behave.
Role in Knowledge Structure	Structures knowledge; serves to organize principles into meaningful frameworks.	Hierarchical; serves as a building block for higher-level core concepts.
Exclusions / Not	Not a list of topics to be learned Not narrow or isolated	Not a superficial fact or procedural step Not narrow or isolated

Following transcript familiarisation, an initial team meeting and joint brainstorming session was held to discuss preliminary impressions of the data. All authors then independently coded two transcripts. Coding was compared during a team meeting in which similarities and discrepancies were discussed, and the level of abstraction was critically examined in relation to the agreed definitions of concepts and principles. These discussions led to the establishment of shared understandings regarding coding boundaries and abstraction levels.

Because clinicians rarely articulated underlying principles explicitly, early coding tended to capture concrete actions, observations, or decision points described in participants’ accounts. Through iterative discussion, the research team examined how these lower-level codes related to shared underlying principles as defined in Table 1. Core concepts were then generated by the full research team by grouping codes that co-occurred across transcripts and jointly explained similar patterns, while meeting pre-agreed upon definitions. This grouping process involved continuous comparison between codes, candidate concepts, and the original data, consistent with Braun and Clarke’s phases of searching for, reviewing, and defining themes [27]. Care was taken to maintain a coherent level of abstraction across concepts and to avoid aggregating conceptually dissimilar codes.

Once the initial coding framework was established, the remaining transcripts were coded independently by each researcher. The team met regularly to compare interpretations, discuss newly emerging codes, and refine conceptual boundaries. Consensus on coding, clustering, and conceptual labels was reached through in-depth discussion among all four authors until shared interpretations were achieved.

Throughout the analytic process, the risk of overinterpretation was actively monitored. The team repeatedly returned to the transcripts to ensure that emerging concepts were firmly grounded in participants’ descriptions. Interpretations were challenged within the group, alternative explanations were considered, and reflexive discussions were held regarding potential researcher bias, particularly given the authors’ professional backgrounds in educational settings. Coding continued until data saturation was achieved and no new core concepts emerged. This reflexive and transparent process strengthened the rigor and credibility of the analysis.

## Results

### Participant characteristics

Table 2 presents a summary of participating clinicians and the characteristics of each consultation serving as the stimulus for their recall session. Clinical experience of the 10 participants ranged from 13 to 36 years. Three participants were employed within a large public hospital, while the remaining 6 were employed within the private sector, representing three separate companies. Stimulus data was collected from a wide range of clinical consultations involving clients whom the participating clinicians had not previously met (*n* = 5) and clients whom the participant had previously met (*n* = 5). Both prosthetic (*n* = 4) and orthotic (*n* = 6) consultations were included. Recall sessions ranged between 42 and 69 min (mean = 56).

**Table 2:** Participating clinicians, including details regarding the consultation that was recorded and used as the recall stimulus

Years experience of clinician	Private/public employer	New or returning client	Prosthesis or orthosis	Reason for consultation
13	Private	Returning client	Prosthesis	Fitting of transtibial prosthesis
15	Private	New client	Orthosis	Ankle-foot orthosis to address drop foot -complication of chemotherapy treatment
20	Private	Returning client	Orthosis	Footwear and ankle-foot-orthosis - Lower extremity weakness following stroke + heel pain
36	Private	Returning client	Prosthesis	Fitting of transtibial prosthesis – recent post-operative amputation
16	Private	New client	Prosthesis	Assessment and casting/scanning for a new transradial prosthesis
36	Private	New client	Prosthesis	Assessment and casting for a new transfemoral prosthesis
19	Private	New client	Orthosis	Lower extremity weakness secondary to Multiple Sclerosis
20	Public	Returning client	Orthosis	Fitting of a knee-ankle-foot-orthosis for detached quadriceps
18	Public	Returning client	Orthosis	Assessment and fit of Cervical orthosis for client with Motor Neurone Disease
23	Public	New client	Orthosis	Assessment and casting for new ankle-foot-orthosis for a clients with a spinal cord injury who presented with a broken device

### Thematic analysis

Quotes representing instances where clinicians applied core principles were coded by the researchers. For example, a participant’s reflection about their client, whom they expected would show more ‘foot-slap’ after walking for a prolonged period, was interpreted as applying principles related to muscle fatigue. As such, the quote was coded under the principle, “Fatigue reduces muscle force and alters neuromuscular control”. Table 3 presents an example of how core principles and concepts were generated from transcript data. A full list of principles and concepts, along with representative quotes, is available as Supplementary File 1. All quotes have been translated from Swedish for the purposes of this publication.

**Table 3:** Example of coding process

Quote	Core principle	Core concept
*Here you can see. Here comes the knee quite clearly. And then to divide it up; Heel strike and knee lift…and then the foot come down*,* like foot flat immediately.*	Phase-specific muscle and joint activity modulate movement	Coordinated Control of Movement
*Yes*,* it’s really to confirm that she has weak plantar flexors. Yes*,* I think she does. …. But she had a really hard time getting up.*	Muscle activation generates movement, insufficient strength compromises control	
*When she gets tired*,* we are going to see a foot slap.*	Fatigue reduces muscle force and alters neuromuscular control	

The thematic analysis identified a total of 11 core concepts, each underpinned by two to eight core principles. Core concepts were organised into four overarching themes reflecting key domains in which core concepts and principles of prosthetic and orthotic practice are enacted: (1) clinical relationships and systems (2), clinical evaluation (3), biomechanics and human movement and (4) device design, materials and context.

### Clinical relationships and systems

Clinical relationships and systems reflects a broad organising theme with three core concepts that help structure clinicians’ reasoning about care delivery within prosthetic and orthotic practice: Clinical & Administrative Systems, Client-Centered Care, and Professional Conduct & Relationships (Table 4).

**Table 4:** Core concepts and underlying principles related to the theme ‘clinical relationships and systems’

	Clinical Relationships & Systems		
Core Concepts	Clinical & Administrative Systems	Client-Centered Care	Professional Conduct & Relationships
Core Principles	Funding, time and resource constraints affect workflow	Active listening informs accurate assessment	Clinicians’ psychological state influenced by client interactions
	Structured workflow ensures safe, efficient care	Clear, empathetic communication builds trust & boosts efficiency	Multidisciplinary collaboration enhances care quality
	Outcome measurement drives accountability and quality improvement	Communication style influences comprehension and participation	
	Referral and funding pathways determine access to P&O devices	Communication is most effective when tailored to the situation and audience	
	Documentation preserves transparency	Communication is influenced by the presence of others	
		Client involvement in decisions strengthens trust, satisfaction, and adherence	
		Empowerment supports independence	
		Education and understanding promotes safe, effective device use	

Clinical & Administrative Systems is a core concept that integrates principles which explain how prosthetic and orthotic care is organised, accessed, delivered, documented, and evaluated. Within this concept, core principles reflect organisational factors, such as funding models, time pressures and resource constraints that shape the actions and decisions available to clinicians. They also explain how administrative processes can support consistency, transparency and accountability across settings.

“*They [health care clinic] receive funding for prescribing assistive devices*,* but they are very restrictive with the amount*.”

(core principle: Referral and funding pathways determine access to P&O devices)

*What has helped us is SwedeAmp [quality register]. XX [name] can see that we have the shortest time from amputation to receiving a prosthesis.*

(core principle: Outcome measurement drives accountability and quality improvement)

*Client-centered Care* is a core concept that describes how clinicians recognise, interpret, and act on client values and goals through effective communication and shared decision-making. The core principles underpinning this concept shape how clinicians elicit and interpret information from their clients and how they adapt their communication strategies to meet individual needs. They also emphasise the importance of client involvement, ownership of care and education to support sustained, safe and effective device use beyond the clinical setting.

I think that the way I work is that I try to be extra clear with them, otherwise there will be a need for adjustments afterwards, they will be unsatisfied because they didn’t fully understand from the beginning.

(core principle: Clear, empathetic communication builds trust & boosts efficiency)

…how she’s going to be able manage this, how she’s going to use it, and that’s that a bit of our role as well. There’s such a big risk that things will go great when we help them and then they come home and they can’t get it together.

(core principle: Education and understanding promotes safe, effective device use)

*Professional Conduct & Relationships* is a core concept that integrates principles explaining how clinicians maintain ethical, responsible and professional behaviour while interacting effectively with clients, colleagues and other members of the healthcare team. Core principles highlight the importance of effective interprofessional collaboration and clinicians’ awareness of how client encounters may affect their own psychological well-being.

It was the first time I had met someone so close who had obviously tried to take their own life, and I was very affected by that situation.

(core principle: Clinicians’ psychological state influenced by client interactions)

### Clinical evaluation

Clinical evaluation reflects a theme encompassing core concepts that structure clinicians’ reasoning about assessments and underlying pathological processes that may influence decision-making in prosthetic and orthotic practice. This theme captures how clinicians identify and prioritise client needs and how they understand the underlying physiological and pathological factors that constrain function and shape intervention choices. Two core concepts were identified: Needs-based Interventions and Pathophysiology as a Constraint on Body Structure & Function (Table 5).

**Table 5:** Core concepts and underlying principles related to the theme ‘clinical evaluation’

	Clinical Evaluation	
Core Concepts	Needs-Based Interventions	Pathophysiology as a Constraint on Body Structure & Function
Core Principles	Structured assessment identifies the root causes of functional deficits	Concurrent treatments interact to affect progression & outcomes
	Comparative evaluations clarify level of impairment and progress	Secondary complications shape recovery trajectories
	Assessment links impairment to functional performance	Functional and recovery are determined by underlying pathophysiology
	Individual characteristics and preferences shape prescription, care pathways & outcomes	Nerve integrity determines muscle function and control
		Pain emerges from interactions between tissues and the nervous system

Needs-Based Interventions describes how prosthetic and orthotic care is fundamentally guided by clients’ functional requirements, priorities and circumstances, ensuring that assessment and prescription are aligned with underlying impairments rather than surface-level presentation alone. Within this core concept, principles emphasise the importance of structured and comparative assessment to distinguish underlying impairments from secondary impairments and to evaluate change over time.

I’m thinking about swelling. Because I know she was quite swollen last time she was with me, so I really just want to check it out a little bit to make sure it hasn’t gotten any worse or different from what I saw when she was here last time.

(core principle: Comparative evaluations clarify level of impairment and progress)

In this core concept, needs were not determined solely through physical assessment. As reflected in a further core principle, individual characteristics, values, and preferences also influenced how needs are interpreted and how intervention pathways are selected.

*The first time he came*,* he didn’t want anything at all*,* so I said yes*,* but then we’ll call the doctor… Because he wanted to have surgery.*

(core principle: Individual characteristics and preferences shape prescription, care pathways & outcomes)

The core concept, *Pathophysiology as a Constraint on Body Structure & Function* describes how underlying biological, neurological, and musculoskeletal changes shape or limit functional capacity, influencing recovery trajectories, device use, and clinical decision-making in prosthetic and orthotic care. Within this concept, core principles emphasise the importance of understanding how different pathological conditions, secondary complications, and concurrent treatment affect function, control and anticipated change over time.

*It is like in some way to understand if this is something we need to have a plan for because it is going to get better*,* or if it is a chronic problem.*

(core principle: Function and recovery are determined by underlying pathophysiology)

### Biomechanics and human movement

Biomechanics and human movement reflects a theme encompassing core concepts that structure clinicians’ reasoning about how mechanical forces, tissue properties, and neuromuscular control interact to produce movement and stability. This theme captures how clinicians understand the generation, transmission, and regulation of forces within the human body, as well as how these forces influence tissue response, movement patterns, and device-user interaction. Three core concepts were identified: Load Transfer to the Human Body, Tissue Adaptation and Remodelling, Coordinated Control of Movement, (Table 6)

**Table 6:** Core concepts and underlying principles related to the theme ‘biomechanics and human movement’

Biomechanics and Human Movement			
Core Concepts	Load Transfer to the Human Body	Tissue Adaptation and Remodeling	Coordinated Control of Movement
Core Principles	Tissue stress is determined by bone geometry and surrounding soft tissues	Disuse leads to atrophy and functional decline	Joint range of motion influences movement patterns and performance
	Concentrated stress leads to tissue damage, even load distribution protects vulnerable structures	Sustained shortening of muscles alters tissue architecture, resulting in contractures	Movement patterns arise from coordinated muscle-joint activity and adapt to changing constraints
	Stress in tissues is determined by force and area over which it is distributed	Tissues remodel in response to mechanical stress	Segment alignment shapes movement by altering force transmission and joint interactions
	Shear and compressive stresses combine to determine tissue adaptation & dagage risk	Tissue adaptation and injury risk are dependent on the magnitude, duration & frequency of mechanical stress	Phase-specific muscle and joint activity modulates movement
	Tissues have distinct mechanical thresholds & respond differently to load		Muscle activation generates movement, insufficient strength compromises control
	Excessive and repeated loading impairs tissue integrity		Fatigue reduces muscle force and alters neuromuscular control
	Lever systems modulate force transmission		
	Movement and stability emerge from the coordination of forces acting on the body		

Load Transfer to the Human Body is a core concept that integrates principles explaining how forces applied to biological tissues are transmitted, absorbed, and tolerated. Within this concept, core principles focus on how mechanical stress arises in tissues, how it is shaped by underlying bone geometry and soft tissue composition, and how stress concentration or dispersion contributes to tissue adaptation or damage.

*So she asked “why is it so high there and so low there?” [AFO with anterior shell] and then I answer yes*,* but it is so high in the front only because here is the shin bone. We need it to distribute the pressure over a larger area.*

(core principle: Stress in tissues is determined by force and area over which it is distributed)

Tissue Adaptation and Remodelling describes how biological tissues modify their structure in response to mechanical, metabolic, and functional demands. Within prosthetic and orthotic care, this core concept explains how tissue adaptation influences movement capacity, device tolerance, and susceptibility to injury. Core principles emphasise the effects of disuse, sustained muscle shortening, and prolonged or excessive mechanical loading on tissue architecture and functional capacity, as well as the potential for unfavourable remodelling or injury.

*I anticipated a flexion contracture and then I expected a certain degree of muscle weakness. Now he was much weaker than I expected.*

(core principle: Sustained shortening of muscles alters tissue architecture, resulting in contractures)

Coordinated Control of Movement, describes how functional movement arises through the dynamic interaction of joints, muscles, and skeletal segments, adapting to task demands, environmental constraints, and individual anatomical characteristics. Within this core concept, principles emphasise that movement patterns are not generated by isolated structures but emerge from coordinated muscle–joint interactions in which joint mobility, force production, and segment alignment collectively shape how movement is produced and controlled.

*She wants to rotate inwards*,* supinate a little as well*,* even if she ins’t a typical stroke. So I think that there is some muscle tone and I want to clarify if it is there and that there is not something more.*

(core principle: Movement patterns arise from coordinated muscle-joint activity and adapt to changing constraints)

### Device design, materials and cantext

Device design, materials, and context reflects a theme encompassing core concepts that structure clinicians’ reasoning about how prosthetic and orthotic devices interact with users and the surrounding environment. This theme captures how clinicians understand the interplay between device design, mechanical properties, material characteristics, and contextual factors, and how these interactions influence device performance, user experience, and safety. Three core concepts were identified: Function as an Interaction of Device, User, and Environment, Mechanical Properties as Determinants of Device Behaviour, and User, Design and Material Constraints in Device Manufacture (Table 7).

**Table 7:** Core concepts and underlying principles related to the theme ‘Device design, materials and environment

	Device Design Materials and Context		
Core Concepts	Function as an Interaction of Device, User & Environment	Mechanical Properties as Determinant of Device Behaviour	User, Design & Material Constraints in Device Manufacture
Core Principles	Functional independence depends on person-environment interactions	Component configuration affects mechanical response	Client characteristics & preferences influence manufacturing methods
	Device effectiveness depends on task demands	Material properties govern how devices deform, transmit force and store energy	Component selection is constrained by clients’ body structure, load tolerance, and functional demands
	Device design, including materials and structure, is dictated by functional requirements	System-level mechanical interactions govern real-world device performance	Material properties impose health and safety constraints during device production
	Outcomes improve when device-user-environment interactions are tailored to individual needs	Elastic elements store and return energy, shaping efficiency and dynamic response	Material behaviour during manufacturing is governed by intrinsic mechanical, thermal & other properties
	Iterative assessment of device, user & environment informs adjustments that optimise function	Force distribution depends on component geometry and material properties	
	Effective coupling between body and device improves functional performance	Material behaviour under repeated or prolonged load affects device performance	
		Flexural rigidity and compliance influence movement and stability	

Function as an Interaction of Device, User & Environment describes how functional performance emerges from the dynamic interplay between the individual, their prosthesis or orthosis, and the surrounding environment. Within this core concept, principles emphasise that functional independence and performance are maximised when device–user–environment interactions are tailored to individual needs, assessed iteratively, and refined to ensure an effective coupling between the body and the device.

*It’s very good. Almost too good [model of prosthetic foot]. You can use it quite far up in activity level. It’s probably better than that*,* it’s just not a low-activity foot*,* many people use it even further up when they are active.*

(core principle: Device effectiveness depends on task demands)

*This is very good because once they get to the walking school*,* you may want to adjust up and down and then replace it with a fixed tube.*

(core principle: Iterative assessment of device, user & environment informs adjustments that optimise function)

Mechanical Properties as Determinant of Device Behaviour describes how the configuration, materials, and geometry of prosthetic and orthotic devices govern their mechanical responses during use. This core concept facilitates understanding of how devices deform, transmit and store force, as well as distribute load. Core principles underlying this concept help clinicians to anticipate device behaviour in real-world contexts and to optimise design to support functional performance, movement efficiency, and stability. Principles cover issues such as how material properties and component configuration affects transmission of force and energy storage, as well as the effects of device geometry on force distribution.

*It is carbon fibre*,* gives a little energy back. Yes absolutely*,* pretty flexible as well.*

(core principle: Elastic elements store and return energy, shaping efficiency and dynamic response)

User, Design and Material Constraints in Device Manufacture describes how client-specific factors and material properties shape decisions related to the manufacture of prosthetic and orthotic devices. Within this core concept, principles emphasise that manufacturing approaches are guided by client characterises, functional requirements, material properties and occupational health and safety. Understanding the behaviour of different materials allows clinicians to select, and safely use, components and manufacturing methods that are compatible with user needs and address potential limitations of specific production methods.

*Fiberglass*,* carbon fiber…. I like fiberglass because it’s really easy to wet*,* to get the resin in.*

(core principle: Material behaviour during manufacturing is governed by intrinsic mechanical, thermal & other properties)

## Discussion

The core concepts and principles of prosthetic and orthotic practice identified in this study are grounded in empirical data and represent an exploratory, practice-informed framework intended to contribute to ongoing discussions in prosthetic and orthotic eduction. They are proposed as a resource to support curricular and course development and may inform, rather than prescribe, the future development of standards documents for prosthetic and orthotic education. As a guiding framework, they may assist educators in organising curricular content and support students in structuring their learning.

The authors do not advocate the use of these concepts and principles as a means of standardising or homogenising educational programs. Rather, we recommend that they be used to map, organise, and frame course and curriculum content in ways that are relevant and responsive to the specific context and circumstances of each educational institution. Furthermore, the concepts and principles should not be regarded as comprehensive or exhaustive, they are offered as a starting point for the continued, iterative development of a practice-informed set of core concepts and principles for the discipline of prosthetics and orthotics.

Concept-based curricula and teaching are gaining increasing traction in health professions education. As students are required to manage an expanding body of knowledge within increasingly complex health systems, concept-based curricula have been proposed as a paradigm shift, moving away from an emphasis on isolated facts and shifting toward organising ideas that support knowledge integration and application [30, 31].

Educators seeking to develop a concept-based approach in prosthetics and orthotics education can utilise the findings of this study by embedding the identified core concepts and principles centrally within curriculum design and aligning teaching activities and assessments with these concepts rather than discrete content topics. For example, in a concept-based prosthetics and orthotics curriculum, the core concept *Load Transfer to the Human Body* could function as a central organising idea across a course or module. Learning activities could be structured around this concept using case-based problems, design critiques, or simulation scenarios in which students are required to analyse how loads are transferred between a specific prosthetic or orthotic device and the user in different clinical contexts. Rather than teaching forces, tissue tolerance, and device fitting as isolated topics, these content areas can be introduced and revisited as principles that inform and deepen understanding of the core concept.

Assessment tasks could then require students to apply this conceptual understanding, for instance by justifying prosthetic or orthotic design decisions in relation to mechanical stress, load distribution, and tissue response, either through written case analyses, oral examinations, or practical assessments. Reflective learning activities could further support students in articulating how the core concept enables them to approach unfamiliar or novel situations, such as working with a new client population or adapting a device to an atypical clinical presentation. In this way, the core concept provides a coherent framework that supports integration, transfer, and clinical reasoning across learning contexts.

The number of core concepts reported across disciplines was recently reviewed by Etukakpan et al. [13]. Among the 30 publications analysed, most studies (*n* = 16) reported between 3 and 10 core concepts, whereas fewer identified either a substantially larger set (10–50 concepts; *n* = 5) or an extensive list exceeding 50 concepts (*n* = 9). The present study identified 11 core concepts, placing our findings slightly above the most reported range. The wide variation in the number of reported core concepts across studies likely reflects a lack of consensus regarding the definition and intended scope of the terms “core concept” and “core principle”. Some authors appear to aggregate broad, organizing ideas [15], while others include more granular or content-specific elements, blurring the distinction between concepts and principles [32, 33]. By explicitly distinguishing core concepts as broad, enduring constructs and core principles as their explanatory foundations, the present study makes a clear distinction between the two terms, which we believe contributes to greater coherence and offers a potential pathway toward greater consensus in future work.

This study represents the first to use a stimulated recall method to identify core concepts for a discipline. Previous work has relied on expert group techniques, discussion workshops, the first round of a Delphi process or document analyses to generate an initial set of core competencies [13]. We argue that the stimulated recall approach offers a distinct methodological advantage by enabling access to clinicians’ reasoning, decision-making processes, and judgments in practice. As such, it provides a practice-informed set of core concepts and their underlying principles, rather than relying solely on expert consensus.

The present set of core concepts and principles represents the first phase in the development of a discipline-specific framework for the prosthetics and orthotics profession. As noted by Etukakpan et al. [13], the next phase of development involves refinement of this initial set, a process for which a range of methodological approaches has been employed. Building on these precedents, we recommend an initial document analysis to map the identified core concepts and principles against relevant prosthetic and orthotic education standard documents [4–7, 34], followed by a Delphi process to establish broader expert consensus [35].

### Limitations

This study has several limitations that should be considered when interpreting the findings. Core concepts and principles were generated from empirical data collected from a purposive sample of ten highly experienced clinicians consulting a client sample of limited breadth. While this expert-focused approach was well-suited to evaluating advanced clinical reasoning, it may have resulted in a limited perspective on the full range of clinical practice. Core concepts and principles that are more relevant to novice clinicians or emerging areas of practice would likely have been overlooked.

The use of thematic analysis in this study resulted in core concepts being represented as discrete, non-overlapping analytic categories. While this approach supported clarity and interpretability, it may have constrained the representation of the underlying conceptual structure as, in practice, core principles may well underpin more than one core concept. Future research could employ alternative or complementary analytic approaches to explore how core principles interact across concepts, including mapping shared principles, examining hierarchical or networked relationships, and investigating how principles are mobilised differently across clinical contexts.

This study was conducted within the Swedish context, where regulatory frameworks, educational pathways, and models of care differ from those in other settings. As a result, it is likely that some core concepts and principles were not captured. These limitations underscore the need for further refinement and validation of this framework across a broader range of practitioners, practice contexts, and international settings.

In addition to the sampling limitations described above, several methodological considerations should be acknowledged. Core concepts and principles were interpreted and generated solely by the research team from the stimulated recall data. Clinicians did not provide feedback on the analytic process or the resulting framework, and therefore, the identified concepts reflect the researchers’ interpretations of the clinicians’ reasoning rather than co-developed or validated constructs. Future work could involve member checking or expert review to refine and validate these findings. Limitations of the video-assisted stimulated recall must also be acknowledged. Although this method supports reflection on tacit clinical reasoning, recall remains retrospective and may be influenced by post-hoc rationalisation, social desirability, or researcher prompting.

## Conclusion

This study used a qualitative, video-assisted stimulated recall method to identify the core concepts and principles guiding Swedish certified prosthetists and orthotists during routine clinical consultations. Analysis of the recall transcripts identified 11 core concepts, each underpinned by between two to eight principles. The identified concepts and principles provide a practice-informed framework to inform the development of concept-based curricula and to support the design of teaching, learning, and assessment approaches that prioritise knowledge integration, transfer, and clinical reasoning over isolated or fragmented content. While further refinement and validation are required, this study establishes an empirically grounded foundation for ongoing work toward a practice-informed set of core concepts and principles for the prosthetics and orthotics profession.

## Supplementary Information

Supplementary Material 1.

## Data Availability

The transcripts generated and analysed during the current study are not publicly available as they are in Swedish, but are available from the corresponding author upon reasonable request.

## References

[1] Orthotics Prosthetics Canada (OPC). OPC scope of practice 2017 [5th April 2023]. Available from: chrome-extension://efaidnbmnnnibpcajpcglclefindmkaj/https://www.opcanada.ca/common/Uploaded%20files/OPC_PDF/Scopes_Of_Practice/Scope-of-Practice-2-21.pdf.

[2] Ash S, O’Connor J, Anderson S, Ridgewell E, Clarke L. A mixed-methods research approach to the review of competency standards for orthotist/prosthetists in Australia. Int J Evid Based Healthc. 2015;13(2):93–103. 26057653 10.1097/XEB.0000000000000038

[3] Ramstrand N, Ramstrand S. Competency standards for newly graduated prosthetist/orthotists in Sweden. Prosthet Orthot Int. 2018;42(4):387–93. 29775171 10.1177/0309364618774056

[4] The Australian Orthotic Prosthetic Association. Entry level competency standards for Australian orthotist/prosthetists. Melbourne, Australia; 2014.

[5] International Society for Prosthetics and Orthotics. Education Standards for Prosthetic/ Orthotic Occupations: ISPO. Available from: https://www.ispoint.org/wp-content/uploads/2022/02/ispo_standards_nov2018_sprea.pdf. Accessed 11 Jan 2026.

[6] The British Association of Prosthetists and Orthotists (BAPO). Standards for best practice 2013 [5th April 2023]. Available from: chrome-extension://efaidnbmnnnibpcajpcglclefindmkaj/https://www.bapo.com/wp-content/uploads/2019/06/BAPO-Standards-Best-Practice-2018-update.pdf.

[7] CAAHEP. Standards and Guidelines for the Accreditation of Educational Programs in Orthotics and Prosthetics. Seminole, FL: Commission on Accreditation of Allied Health Education Programs,; 2021.

[8] Schaefer JE, Michael J. Core concepts: views from physiology and neuroscience. Front Educ. 2024;9:2024.

[9] Michael J, McFarland J. The core principles (big ideas) of physiology: results of faculty surveys. Adv Physiol Educ. 2011;35(4):336–41. 22139767 10.1152/advan.00004.2011

[10] Tangalakis K, Julien BL, Lexis L, Hryciw DH, Thomas CJ, Husaric M, et al. Mapping the core concepts of physiology across Australian university curricula. Adv Physiol Educ. 2023;47(3):411–8. 37141433 10.1152/advan.00139.2022

[11] Chen A, Phillips KA, Schaefer JE, Sonner PM. Community-Derived Core Concepts for Neuroscience Higher Education. CBE Life Sci Educ. 2023;22(2):ar18. 36862801 10.1187/cbe.22-02-0018 PMC10228273

[12] Tweedie J, Pelly F, Wright H, Palermo C. Exploring the adoption of concept-based curricula: insights from educators and implications for change. Adv Health Sci Educ Theory Pract. 2025;30(1):223–37. 38829549 10.1007/s10459-024-10346-y PMC11925998

[13] Etukakpan AU, Waldhuber MG, Janke KK, Netere AK, Angelo T, White PJ. Core concept identification in STEM and related domain education: a scoping review of rationales, methods, and outputs. Front Educ. 2025;10:2025.

[14] Mitchell I, Keast S, Panizzon D, Mitchell J. Using ‘big ideas’ to enhance teaching and student learning. Teachers Teach. 2017;23(5):596–610.

[15] Michael J, Modell H, McFarland J, Cliff W. The core principles of physiology: what should students understand? Adv Physiol Educ. 2009;33(1):10–6. 19261754 10.1152/advan.90139.2008

[16] Chen A, Phillips KA, Schaefer JE, Sonner PM. The Development of Core Concepts for Neuroscience Higher Education: From Beginning to Summer Virtual Meeting Satellite Session. J Undergrad Neurosci Educ. 2022;20(2):A161–5. 38323056 10.59390/GHOR4737 PMC10653233

[17] Meade P, McMeniman M. Stimulated recall — An effective methodology for examining successful teaching in science. Australian Educational Researcher. 1992;19(3):1–18.

[18] Zhai X, Chu X, Wang M, Tsai C-C, Liang J-C, Spector JM. A systematic review of Stimulated Recall (SR) in educational research from 2012 to 2022. Humanit Social Sci Commun. 2024;11(1):489.

[19] Sinnott C, Kelly MA, Bradley CP. A scoping review of the potential for chart stimulated recall as a clinical research method. BMC Health Serv Res. 2017;17(1):583. 28830405 10.1186/s12913-017-2539-y PMC5567630

[20] Burden S, Topping A, O’Halloran C. The value of artefacts in stimulated-recall interviews. Nurse Res. 2015;23(1):26–33. 26365073 10.7748/nr.23.1.26.e1324

[21] Dewey J. How we think: a restatement of the relation of reflective thinking to the educative process. DC Health, editor. Boston: D.C. Health & Co; 1933.

[22] Blackhall VI, Walker KG, Whiteley I, Wilson P. Use of head camera-cued recall and debrief to externalise expertise: a systematic review of literature from multiple fields of practice. BMJ Simul Technol Enhanc Learn. 2019;5(3):121–9. 10.1136/bmjstel-2018-000341 PMC8936570 35514944

[23] Labonte R, Robertson A. Delivering the Goods, Showing Our Stuff: The Case for a Constructivist Paradigm for Health Promotion Research and Practice. Health Educ Q. 1996;23(4):431–47. 8910022 10.1177/109019819602300404

[24] Lyle J. Stimulated Recall: A Report on Its Use in Naturalistic Research. Br Edu Res J. 2003;29(6):861–78.

[25] van Braak M, de Groot E, Veen M, Welink L. Eliciting tacit knowledge: The potential of a reflective approach to video-stimulated interviewing. Perspect Med Educ. 2018;7(6)386–93. 10.1007/s40037-018-0487-9 PMC6283779 30446951

[26] Kosicki GM. Encyclopedia of Survey Research Methods. In: Lavrakas P, editor. Encyclopedia of Survey Research Methods. Thousand Oaks, Ca.: SAGE; 2008.

[27] Braun V, Clarke V. Reflecting on reflexive thematic analysis. Qualitative Res Sport Exerc Health. 2019;11(4):589–97.

[28] Ramstrand N, Mussa A, Gigante I. Factors influencing satisfaction with prosthetic and orthotic services - a national cross-sectional study in Sweden. Disabil Rehabil. 2024;46(25):6213–20. 38400691 10.1080/09638288.2024.2319342

[29] McFarland J, Michael J. Reflections on core concepts for undergraduate physiology programs. Adv Physiol Educ. 2020;44:626–31. 32990460 10.1152/advan.00188.2019

[30] Brussow JA, Roberts K, Scaruto M, Sommer S, Mills C. Concept-Based Curricula: A National Study of Critical Concepts. Nurse Educ. 2019;44(1):15–9. 29474344 10.1097/NNE.0000000000000515 PMC6276859

[31] Giddens JF, Brady DP. Rescuing nursing education from content saturation: the case for a concept-based curriculum. J Nurs Educ. 2007;46(2):65–9. 17315564 10.3928/01484834-20070201-05

[32] Tweedie J, Palermo C, Wright HH, Pelly FE. Using document analysis to identify core concepts for dietetics: The first step in promoting conceptual learning. Nurs Health Sci. 2020;22(3):675–84. 32166858 10.1111/nhs.12712

[33] Qian Y, Wang Y, Wen J, Wu S, Zhang J. One Hundred Core Concepts in Chemistry and Upper-Secondary School Teachers’ and Students’ Chemistry Conceptual Structures. J Baltic Sci Educ. 2023;22(3):493–505.

[34] CAAHEP. Standards and Guidelines for the Accreditation of Educational Programs in Orthotics and Prosthetics. Commission on Accreditation of Allied Health Education Programs; 2021. https://cdn.prod.websitefiles.com/5f466098572bfe97f28d59df/603da2518a8d7dd3fccdd62b_OPStandardsGuidelines2017.pdf. Accessed 11 Jan 2026.

[35] Barrett D, Heale R. What are Delphi studies? Evid Based Nurs. 2020;23(3):68. 32430290 10.1136/ebnurs-2020-103303

